# Esrrb Regulates Specific Feed-Forward Loops to Transit From Pluripotency Into Early Stages of Differentiation

**DOI:** 10.3389/fcell.2022.820255

**Published:** 2022-05-16

**Authors:** Amin R. Mazloom, Huilei Xu, Jaume Reig-Palou, Ana Vasileva, Angel-Carlos Román, Sonia Mulero-Navarro, Ihor R. Lemischka, Ana Sevilla

**Affiliations:** ^1^ Department of Pharmacology and Systems Therapeutics, Icahn School of Medicine at Mount Sinai, New York, NY, United States; ^2^ Department of Cell Biology, Physiology and Immunology, Faculty of Biology, University of Barcelona, Barcelona, Spain; ^3^ Center for Radiological Research, Columbia University, New York, NY, United States; ^4^ Department of Biochemistry, Molecular Biology and Genetics, University of Extremadura, Badajoz, Spain; ^5^ Department of Developmental and Regenerative Biology, Icahn School of Medicine at Mount Sinai, New York, NY, United States; ^6^ Black Family Stem Cell Institute, Icahn School of Medicine at Mount Sinai, New York, NY, United States; ^7^ Institute of Biomedicine of the University of Barcelona (IBUB), Barcelona, Spain

**Keywords:** estrogen related receptor beta, feed-forward loops, MicroRNAs, mouse embryonic stem cells, network dynamic analysis

## Abstract

Characterization of pluripotent states, in which cells can both self-renew or differentiate, with the irreversible loss of pluripotency, are important research areas in developmental biology. Although microRNAs (miRNAs) have been shown to play a relevant role in cellular differentiation, the role of miRNAs integrated into gene regulatory networks and its dynamic changes during these early stages of embryonic stem cell (ESC) differentiation remain elusive. Here we describe the dynamic transcriptional regulatory circuitry of stem cells that incorporate protein-coding and miRNA genes based on miRNA array expression and quantitative sequencing of short transcripts upon the downregulation of the Estrogen Related Receptor Beta (Esrrb). The data reveals how Esrrb, a key stem cell transcription factor, regulates a specific stem cell miRNA expression program and integrates dynamic changes of feed-forward loops contributing to the early stages of cell differentiation upon its downregulation. Together these findings provide new insights on the architecture of the combined transcriptional post-transcriptional regulatory network in embryonic stem cells.

## Introduction

Naïve pluripotent embryonic stem cells (ESCs) and epiblast stem cells (EpiSCs) constitute different developmental stages, mimicking the pre- and the post-implantation events during the embryo development respectively (Semi and Takashima, 2021). The complex molecular mechanisms governing this cellular stage transition are orchestrated by fluctuating levels of pluripotency transcription factors and wide-range modelling of the epigenetic landscape (Sevilla et al., 2021). Recently, microRNAs (miRNAs) have also emerged as important post-transcriptional regulators of cell fate (Leung et al., 2011; Li et al., 2021). In particular, miRNAs have been shown to play a key role in mammalian cell differentiation for proper embryonic development into the three germ layers (Cirera-Salinas et al., 2017; Cui et al., 2021). Additionally, recent studies focused on deconstructing the transcriptional heterogeneity of ESCs, have identified certain differentially expressed miRNAs as key mediators of this transcriptional heterogeneity that facilitate the transitions into different cellular states (Kumar et al., 2014; Chakraborty et al., 2020).

Among the mechanisms to control the ESC miRNA expression program, we find the direct binding of certain transcription factors like Oct4, Sox2, Nanog and Tcf3 to their specific miRNA promoter regions (Marson et al., 2008). These miRNAs or small non-coding RNA molecules with approximately 23 nucleotides (nt) in length, regulate in turn the expression of a large set of genes as well as the pluripotency transcription factors themselves. Their way to modulate the expression of large groups of target genes is by base pairing with complementary sequences in mRNAs to induce mRNA decay and translational repression (Bartel, 2009). From this point of view, the deep understanding of network regulatory functions involves the coordination of several molecular regulatory mechanisms over time.

As key regulators of gene expression, transcription factors (TFs) and miRNAs are able to co-regulate the expression of targets in form of feed-forward loops (FFLs) and feedback loops (FBLs) (Eduati et al., 2012; Macarthur et al., 2012; Bo et al., 2021; Sevilla et al., 2021). These two kind of loops are important motifs in gene regulatory networks, which were initially proposed to describe co-regulation between different TFs on the same target (Kalir et al., 2005; Hirashima et al., 2008). In this regard, we already have been able to identify certain FFLs between Esrrb and Nanog regulating the expression of common target genes (Sevilla et al., 2021). Here, we hypothesize that TFs and miRNAs can co-regulate gene expression in a similar manner when cells transit into early differentiation stages.

In particular, it is well known that an intricate network of miRNAs participates in the regulation of the ESC cell cycle, ESC self-renewal and reprograming and ESC differentiation (Li et al., 2017). In the pluripotency state, the miRNA 290–295 cluster accounts for more than 60% of the miRNA population (Yuan et al., 2017). Indeed, components of this cluster, such as the mmu-mir-294 promote pluripotency by regulating a subset of c-Myc target genes through feed-forward loops and directly upregulating pluripotency-associated genes such as Lin 28 (Hanina et al., 2010). In this regard, work pioneered by Marson and others also proposed the presence of incoherent feed-forward loops among Oct4, Sox2, Nanog and Tcf3 and the miR 290–295 cluster (Marson et al., 2008).

To extend these studies, we have centred this study in mapping the direct miRNA targets of the Estrogen related receptor beta, (Esrrb), and observed their miRNA dynamic changes once integrated into gene regulatory networks with their gene target transcripts. Studying the dynamic changes of these feedforward motifs participating miRNAs will give us a better understanding on this stem cell transition upon Esrrb downregulation.

Esrrb was discovered as a key pluripotency transcription factor (TF) with Pou5f1, Nanog and Sox2 from loss of function studies (Ivanova et al., 2006). Results from its depletion evidenced loss of pluripotency and certain cellular commitment towards epiblast-derived lineages, such as mesoderm and neuroectoderm (Ivanova et al., 2006; Festuccia et al., 2018). It has been shown that Esrrb is among the key TFs present in ESC while absent in more mature epiblast-derived stem cells (EpiSC) (Hutchins et al., 2013). In ESCs, Esrrb and Sox2 positively co-regulate at the transcriptional level, certain pluripotent genes and co-binding with Oct4 is capable to activate Nanog promoter (van den Berg et al., 2008).

Interestingly, further observations of our previous studies in protein/mRNA content (Lu et al., 2009), highlighted the important and largely underappreciated role(s) of translational and post-translational regulation in ESC biology. To address this issue in more detail, we have integrated miRNA expression analyses into our regulatory networks providing a more comprehensive view of the stem cell transition towards a differentiation state. We identify a set of feed-forward loops (FFL) where Esrrb (and likely other transcription factors) simultaneously regulate the expression of mRNA coding genes and miRNA genes whose products have been experimentally proved to target these mRNAs. Thus, Esrrb simultaneously regulates protein-coding genes as well as the post-transcriptional machinery for fine-tuning the transition from pluripotency into an early differentiation state. Overall, our results reveal the amazing degree of biological complexity that can be “encoded” even with apparently “simple” regulatory network sub-circuits.

## Materials and Methods

### ES Cell Culture

The murine ESC line with controllable Esrrb expression (Esrrb_R) was constructed and characterized previously (Ivanova et al., 2006), and was maintained as described on irradiated primary mouse embryonic fibroblasts (MEFs). For all experiments, ESCs were cultured on 0.1% gelatin-coated tissue culture plates without feeder cells. To induce differentiation, we plated the cells at a density of 3×10^5^ cells per 10 cm dish and we withdrew Doxycycline (Dox) (1 μg/ml, Sigma) from the media while maintaining all other routine ESC nutrients: D-MEM–High Glucose (Dulbecco’s modified Eagle’s medium-1X-High Glucose) (Gibco^®^, Invitrogen), 15% FBS (Fetal bovine serum) (Hyclone, Thermo Scientific), 100 mM MEM non-essential amino acids, 0.1 mM 2-mercaptoethanol, 1 mM L-glutamine, Penicillin/Streptomycin (Gibco, Invitrogen) and 10^3^ U/ml LIF (Chemicon, Millipore). All cell cultures were maintained at 37°C with 5% CO_2_.

### Alkaline Phosphatase

Alkaline phosphatase activity was examined using Alkaline Phosphatase Staining Kit (Red) from abcam (ab242286) following manufacturer´s instructions.

### Real Time Quantitative PCR (RT-qPCR)

Cells were trypsinized and collected at specific time points. Total RNA was extracted using Trizol Reagent (Invitrogen), column-purified with RNeasy kit (Qiagen) and treated with RNase-free DNase (Qiagen). Total RNA (1 μg) was reverse transcribed using a high-capacity reverse transcription kit (Applied Biosystems). All quantitative PCR analyses were performed using the Fast SYBR Green Master Mix (Applied Biosystems) following the manufacturer’s protocols on the StepOne Plus Real-Time PCR System (Applied Biosystems). Gene-specific primers used for this study are listed in Supplementary Table S1.

### MiRNA qRT-PCR

MiRNA expression levels were quantified in total RNA from cell extracts at days 0, 1, 3 and 5 using Trizol and MiRNase kit (QIAGEN). TaqMan mouse MicroRNA assays (Applied Biosystems) were used to quantify mature miRNA expression levels as previously described (Chen et al., 2005) (Supplementary Table S1). Reverse-transcriptase reactions were performed using the TaqMan MicroRNA Reverse Transcription kit (Applied Biosystems) according to manufacturer´s recommendations. Basically, the reaction contained 10 ng of total RNA, 50 nM reverse transcription (RT) primer, 1x RT buffer, 0.25 mM each of dNTPs, 3.33 U/ml MultiScribe reverse transcriptase and 0.25 U/ml RNase inhibitor. The 7.5 μl reactions were incubated in an Applied Biosystems 9,700 Thermocycler in a 96-well plate for 30 min at 16°C, 30 min at 42°C, and 5 min at 85°C and then held at 4°C. Quantitative real-time PCR was performed using TaqMan universal PCR Master Mix, No AmpErases UNG (Applied Biosystems) on the StepOnePlus Real-Time PCR Systems (Applied Biosystems). The 10 μl PCR mix included 0.67 μl RT product, 1x TaqMan universal PCR Master Mix, and 0.2 mM TaqMan probe. The reactions were incubated in a 96-well plate at 95°C for 10 min, followed by 40 cycles of 95°C for 15 s and 60°C for 1 min. All reactions were run in triplicate. All TaqMan miRNA assays are available through Applied Biosystems. MiRNA expressions were normalized to the expression of U6 probe (Applied Biosystems) as endogenous control.

### Chromatin Immunoprecipitation

Data from the Esrrb ChIP using the PP-H6707-00 antibody, clone H6707 (R&D Systems) was obtained from the GEO database under the accession numbers GSM785839 and GSM785840. See Supplementary Material and our previous work for experimental details (Sevilla et al., 2021).

### Analysis of Mature miRNA Frequencies by Solexa Sequencing

The method for cloning cDNAs from 18–30 nt transcripts was slightly modified from the previously described to allow its use in the Solexa sequencing platform (Illumina) (Lau et al., 2001). Short transcript libraries were generated using size selected RNA. Total RNA at different time points after Esrrb downregulation, was extracted with Qiazol^®^ (Qiagen) with subsequent enrichment for small RNAs (<200 bp) using the miRNeasy^®^ Mini purification kit according to the manufacturers’ instructions for total RNA isolation that includes the small fraction. Extracted RNA (40 µg) was combined with trace amounts of 5′-^32^P-labeled RNA standards (See, Supplementary Table S1). RNA was then fractionated on a 15% polyacrylamide, 8 M urea gel (Bio-Rad). A gel fragment spanning both the 18 nt and 24–26 nt internal standards was excised, and RNA was eluted and ethanol-precipitated in siliconized tubes, with 20 µg of glycogen as carrier. Gel-purified 18–26 nt RNA was incubated with 50 µM pre-adenylylated 3′-adaptor oligonucleotide (Modban), 10X ATP-free ligase Buffer (500 mM Tri-HCl (pH = 7.5–7.6), 100 mM MgCl_2_, 100 mM DTT, 600 μg/ml BSA), and T4 RNA Ligase 2, truncated (New England BioLabs) in a 20 µl reaction at 37°C for 1 h. The T4 RNA ligase reaction was purified on a 15% polyacrylamide, 8 M urea gel (Bio-Rad) by using the ligated forms of the standards as a guide for band excision. RNAs ligated with 3′-adaptor oligonucleotide were eluted and ethanol-precipitated in siliconized tubes, with 20 µg of glycogen. Ligated RNA product was used in a second T4 RNA ligase reaction for the 5′-adaptor oligonucleotide (Solexa Linker). Ligated products were gel-purified, excising the gel fragment spanning the doubly ligated standards. Gel-purified doubly ligated RNA was used in a standard 20 µl RT reaction (SuperScript III, Invitrogen) with the RT BanOne primer. The cDNA was PCR amplified with the 5′and 3′primers, generating products with an extended 3′ adaptor sequence. PCR products were digested with Pme I (NEB) at 37°C for 3 h. DNA fragments ranging from 108–113 bp were isolated from a low-melting point agarose gel. After further phenol extraction and ethanol precipitation, DNA samples were resuspended in Elution buffer (10 mM Tris/0.1% Tween) and then used according to the standard Solexa sequencing protocol (Illumina). Each library was run on one lane of the Solexa sequencer at the Genomic Sequencing Core at Icahn School of Medicine at Mount Sinai. MiRNA-Seq data analysis is described in the Supplementary Material and primer sequences for this analysis can be obtained from (Supplementary Table S1).

### Gene Ontology

Gene ontology analysis was performed using David bioinformatic resources https://david.ncifcrf.gov/tools.jsp (Huang et al., 2008).

### qRT-PCR of Primary miRNAs

qPCR primers were designed using the standard specifications of Primer 3 for real time primer design (Rozen and Skaletsky, 2000). Pri-miR 290–295 expression levels were analysed by SybrGreen quantitative PCR on the StepOnePlus Real-Time PCR system (Applied Biosystems) using specific primers (Supplementary Table S1). Expression levels were calculated relative to actin mRNA levels.

### miRNA Microarray Expression Analysis

RNA from the Esrrb_R rescue clone was extracted with the miRNeasy mini kit (Qiagen) at the different time points. Total RNA (5 µg) from days 1, 3 and 5 after Esrrb downregulation and day 0 as a reference sample were labelled with Hy3™ and Hy5™ fluorescent labels, using the miRCURY™ LNA Array labelling kit (Exiqon, Denmark) according to the manufacturer’s protocol. The labelled samples were mixed pairwise and hybridized to the miRNA arrays printed using miRCURY™ LNA oligo set, fifth generation (Exiqon, Denmark). Analyses were performed in triplicate for a total of 12 microarrays. Each miRNA was printed in duplicate, on code link slides (GE), using Gene Machines Omnigrid 100. Hybridizations were performed overnight at 60°C using the Agilent Hybridization system (SurHyb), after which the slides were washed using the miRCURY™ LNA washing buffer kit (Exiqon, Denmark) following the manufacturer’s protocol. The slides were scanned using an Axon 4000B scanner and image analyses were performed using the Genepix Pro 6.0 software package. MiRNA microarray expression data analysis is depicted in the Supplementary Material.

## Results

### Esrrb Downregulation Changes the miR Transcriptome Landscape

**FIGURE 1:**
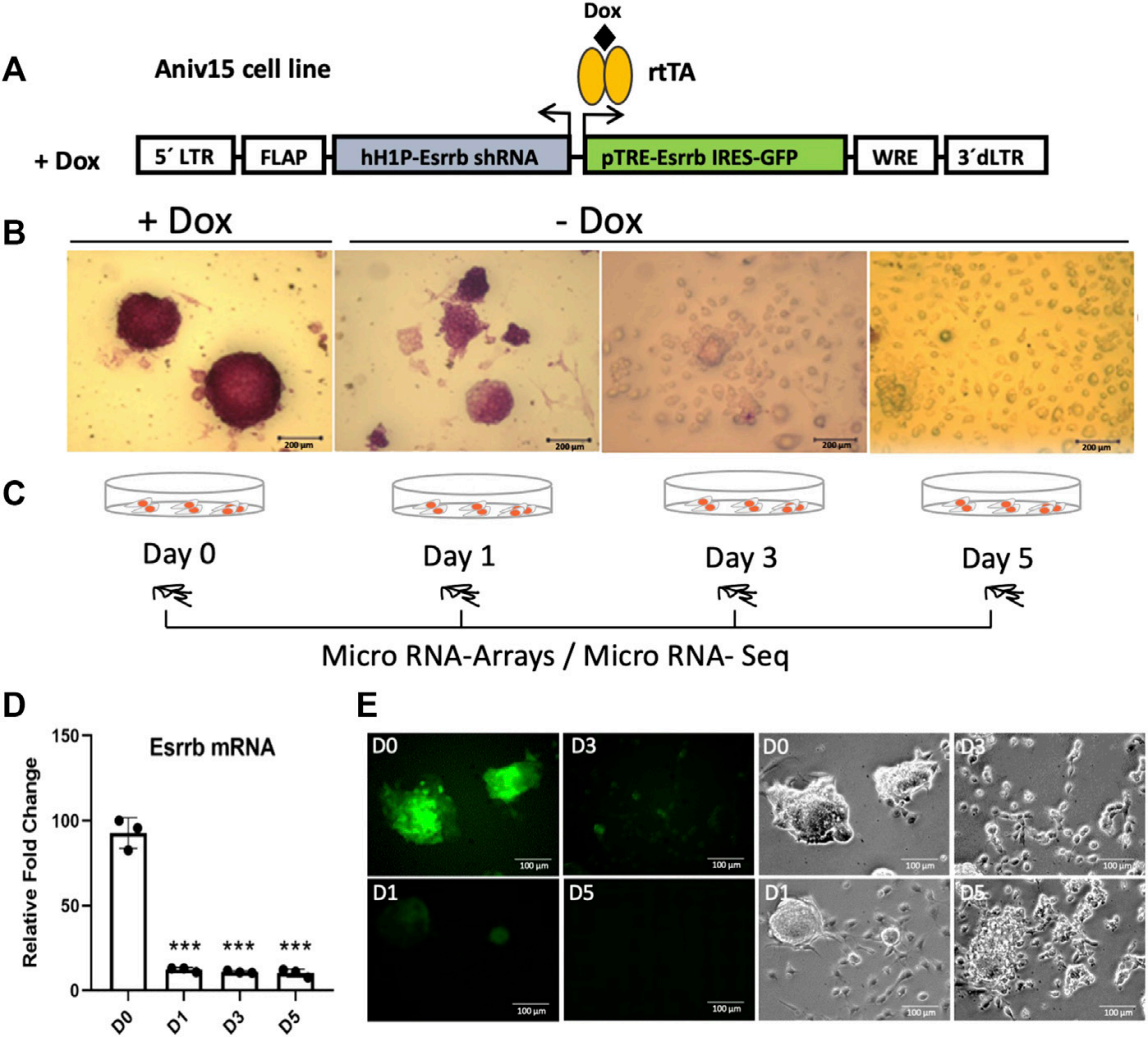
Downregulation of Esrrb induces cell differentiation **(A)** Structure of the lentiviral vector for conditional expression of Esrrb (Ivanova et al., 2006). Endogenous Esrrb is depleted with short hairpin (sh) RNA and complemented by shRNA ¨immune¨ version of Esrrb expressed in a doxycycline (Dox)-dependent manner. Dox withdrawal results in downregulation of the exogenous Esrrb leading to stem cell differentiation. hH1P-Esrrb is the endogenous Esrrb specific shRNA cassette (in light grey); pTRE-Esrrb is GFP-tagged exogenous Esrrb cassette (in green) **(B)** Experimental time series inducing differentiation from day 1 to day 5 by Dox removal and measurement of the alkaline phosphatase activity to confirm stem cell differentiation. Scale bar 200 µm. **(C)** Experimental time course for miRNA gene expression analysis. At day 0, Esrrb is expressed in the presence of Dox; at day 1, 3 and 5 time points, Esrrb is downregulated following Dox withdrawal. **(D)** Gene expression analysis of Esrrb after Dox withdrawal. Endogenous levels of Esrrb mRNA are undetectable due to the constitutive shRNA. Quantitative PCR data confirm the expression of the exogenous form of Esrrb in the presence of Dox (mean ±3 replicates (n = 3)). Significance was tested comparing each day to day 0 using a two-tailed Student´s *t*-test with _***_ *p* < 0.001) **(E)** GFP expression at day 0 in the presence of Doxycycline. Removal of Doxycycline results in the downregulation of the exogenous Esrrb-GFP leading to stem cell differentiation. Scale bars 100 µm.

To explore the Esrrb miRNA specific program, we started analysing miRNA dynamic changes upon its downregulation. For that, we took advantage of the lentiviral/shRNA-based genetic complementation system to deplete Esrrb under serum/Lif conditions (Ivanova et al., 2006; Lee et al., 2012; Sevilla et al., 2021) (Figure 1A). This lentiviral system carries a constitutively expressed shRNA for Esrrb and a TRE-controlled immune-deficient version of Esrrb linked to GFP expression, into transactivator (rtTA)-expressing mouse Ainv15 ESCs. Therefore, in the presence of doxycycline (day 0), Esrrb is expressed but upon doxycycline removal, Esrrb expression downregulates and cells differentiate. Stem cell differentiation at days 1, 3 and 5, was confirmed by the loss of alkaline phosphatase activity in comparison to day 0 (Figure 1B). Using this system, we profiled miRNA expression at day 0, when Esrrb was expressed and at days 1, 3 and 5, where Esrrb downregulation was induced (Figures 1C–E), by exiqon microarrays and miRNA-Seq.

**FIGURE 2:**
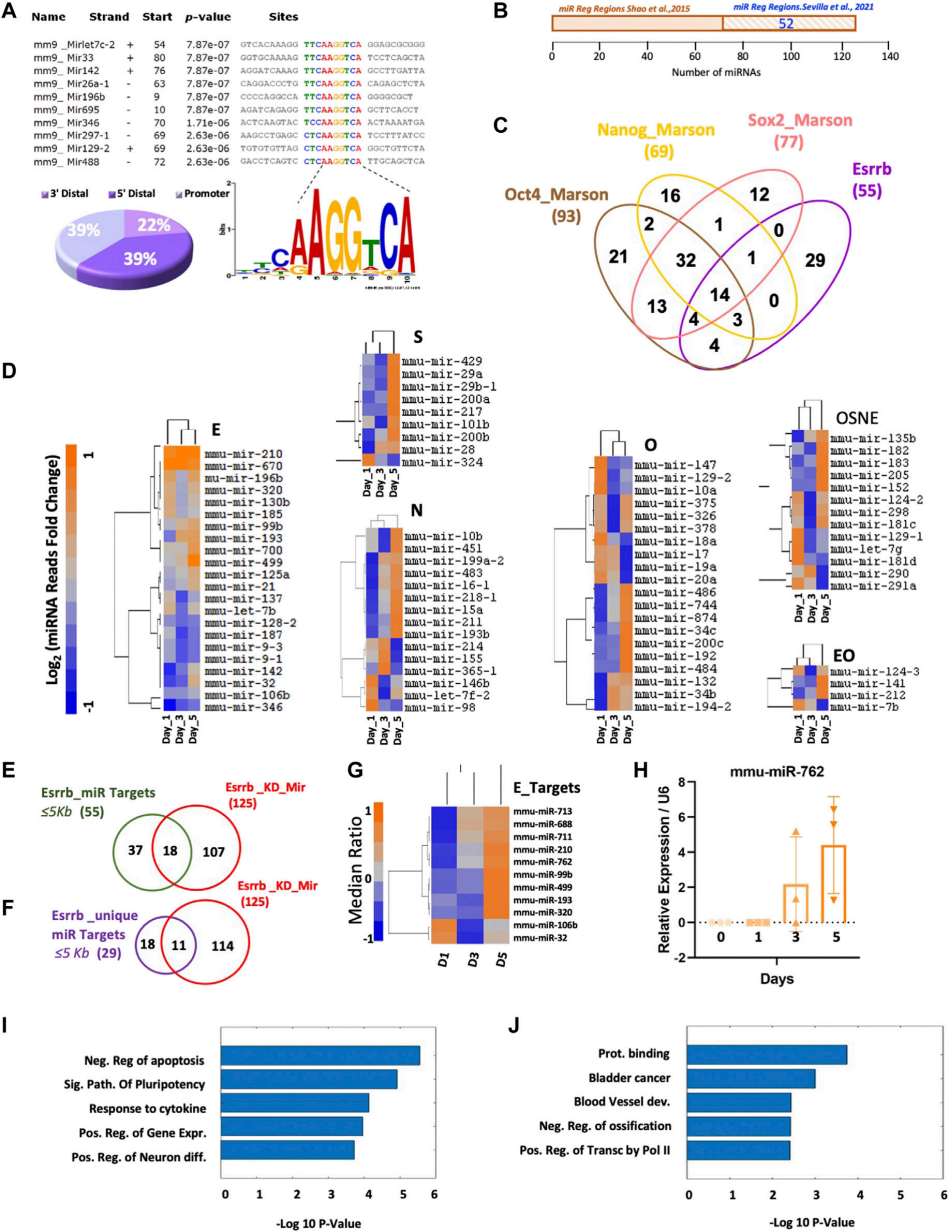
Direct regulation of miRNA expression by Esrrb. **(A)** Motif Esrrb peak distribution at miRNA promoters, (22%) 3′Distal (39%) 5′Distal and (39%) Promoter. **(B)** Venn diagram generated by the Venny 2.1.0 tool https://bioinfogp.cnb.csic.es/tools/venny/(Oliveros, 2007) showing the overlap between the Esrrb miRNA target genes identified by Shao et al. (2015) and the Esrrb miRNA target genes identified in our Esrrb ChIP-Seq data (Sevilla et al., 2021) **(C)** Venn diagram generated by the Venny 2.1.0 tool https://bioinfogp.cnb.csic.es/tools/venny/(Oliveros, 2007) showing the overlaps among miRNAs bound by Esrrb, and those bound by Oct4, Sox2 and Nanog (Marson et al., 2008). Binding sites were identified at ≤5 Kb distance from the transcription start site (TSS) for all four-transcription factors. Numbers in parentheses represent the total number of miR genes bound by each respective TF. **(D)** Dynamic miRNA expression level changes of miRNA genes that are targets of Esrrb (letter E) Oct4 (letter O), Nanog (letter N), Sox2 (letter S), the combination of all four transcription factors (letters OSNE) and the combination of only Oct4 and Esrrb (letters EO) following Esrrb depletion. Analysis and normalization details are described in Material and Methods, Supplementary Material and Supplementary Figure S1. A full list of the miRNA reads differentially expressed can be found in (Supplementary Table S5) **(E)** Venn diagram showing the overlap between the 55 Esrrb miRNA targets and the 125 differentially expressed miRNAs analyzed by microarrays. Anova *p*-Value ≤ 0.05 after Bonferroni correction (Supplementary Table S6) **(F)** Venn diagram showing the overlap between the 29 unique Esrrb miRNA targets and the 125 differentially expressed miRNAs analyzed by microarrays (Supplementary Table S6). Anova *p*-Value ≤ 0.05 after Bonferroni correction. **(G)** Hierarchical clustering of the miRNAs that are specifically regulated by Esrrb, but not by Oct4, Sox2 or Nanog that significantly change in expression after Esrrb knockdown. Expression levels are represented as ratios of the median. **(H)** Relative expression of the mature mmu-mir-762 over the time series. Error bars indicate standard deviation derived from three independent time series experiments (n = 3). **(I)** Bars representing the five top Gene ontology (GO) terms obtained, using David bioinformatics resources https://david.ncifcrf.gov/tools.jsp (Huang et al., 2008), in the set of genes that are directly bound by Esrrb as transcription factor and also modulated by Esrrb-regulated miRNAs. **(J)** GO results from the set of genes that are only modulated by Esrrb-regulated miRNAs.

Surprisingly, in genomic localization analyses, when we incorporate our published Esrrb ChiP-Seq data GSM785839 (Sevilla et al., 2021) to our miRNA analysis, we observed that 39% of Esrrb binding sites are located near miR gene promoter regions and these binding sites contain the previously reported Esrrb binding motif TCAAGGTCA (Chen et al., 2008) (Figure 2A and Supplementary Table S2). Comparison of our Esrrb miRNA targets with the ones previously published showed an overlap of 52 miRNAs (57%) (Shao et al., 2015) (Figure 2B and Supplementary Table S3). Integration of Esrrb miRNAs binding sites with the other core transcription factors, all within ≤5 Kb from the transcription start site (TSS), showed a total of 14 Esrrb miRNA targets that are co-occupied by Esrrb, Oct4, Sox2 and Nanog and 29 that are uniquely bound by Esrrb (Figure 2C and Supplementary Table S4). MiRNA-Seq analysis of the dynamic changes of the miRNAs regulated by each transcription factor independently showed specific miRNAs like mmu-mir-210, mmu-mir-99b, mmu-mir-499, mmu-mir-196b, mmu-mir-320, mmu-mir-106b and mmu-mir-32 among others to be regulated by Esrrb whereas, other miRNAs like mmu-mir-182, mmu-mir-183, mmu-mir -290 and mmu-mir-291a appeared to be regulated by the whole core of transcription factors Oct4, Sox2, Nanog, and Esrrb (OSNE) (Figure 2D, Supplementary Table S5).

Further comparison of the fifty-five Esrrb miRNA targets with the miRNA transcriptome microarray data performed in the Esrrb knockdown time series, revealed significant changes of expression in eighteen out of the fifty-five (Figure 2E, Supplementary Table S6). Notably, eleven out of the eighteen miRNAs identified were uniquely regulated by Esrrb (Figure 2F, Supplementary Table S6). Heatmap showing the miRNA expression values, from our microarray data along the time series, showed that most of them are upregulated (Figure 2G, Supplementary Table S6). Additional experimental confirmation for mmu-mir-762, from three independent experimental time series, confirmed this upregulation as well (Figure 2H).

Next, we explored if the main transcriptional changes upon Esrrb downregulation of these eleven miRNAs were attributed to regulation through the modulation of miRNAs or to a direct control as a transcription factor. For that, we identified the miRNA target genes of these eleven Esrrb regulated miRNAs using the miRNet 2.0 network-based visual analytics for miRNA functional analysis www.mirnet.ca (Chang et al., 2020) and we classified those genes as being regulated by Esrrb as a transcription factor or not based on the presence or absence of peaks at (≤5 Kb from TSS) using the data from our previous publication (Sevilla et al., 2021). Interestingly, gene ontology analysis using David bioinformatic resources https://david.ncifcrf.gov/tools.jsp (Huang et al., 2008) of these eleven Esrrb miRNA target genes showed a significant enrichment in the categories of negative regulation of apoptosis, signaling pathways of pluripotency and positive regulation of gene expression for those genes that are both regulated directly by Esrrb as a transcription factor (≤5 Kb from TSS), and also modulated by Esrrb through the regulation of miRNAs, (Figure 2I and Supplementary Table S7). However, in the set of genes that are only regulated by Esrrb through the modulation of miRNAs, we observed an enrichment in more diverse categories such as protein binding, bladder cancer and blood vessel development (Figure 2J and Supplementary Table S7). These results reinforce the role of Esrrb as a transcription factor not only interacting directly at the promoter region of the genes but also binding at the promoter regions of certain miRNAs whose target genes are also involved in regulating gene expression.

Further analysis of the targets of each miRNA showed that not all the miRNAs regulate the same number of genes. For instance, mmu-mir-688, mmu-mir-711, mmu-mir-99b and mmu-mir-193 have less weight in the Esrrb miRNA regulation as each of them regulate less than 10 target genes. In contrast, mmu-mir-713, mmu-mir-210, mmu-mir-762, mmu-mir-499, mmu-mir-320, mmu-mir-106b, and mmu-mir-320 each of them regulate, up or down, more than 10 target genes. As we can observe, the response is quite specific for each miRNA and each target (Supplementary Figure S2 and Supplementary Table S8).

### Esrrb Contributes With the Core Transcription Factors Oct4, Sox2 and Nanog in the miR 290–295 Cluster Regulation

**FIGURE 3:**
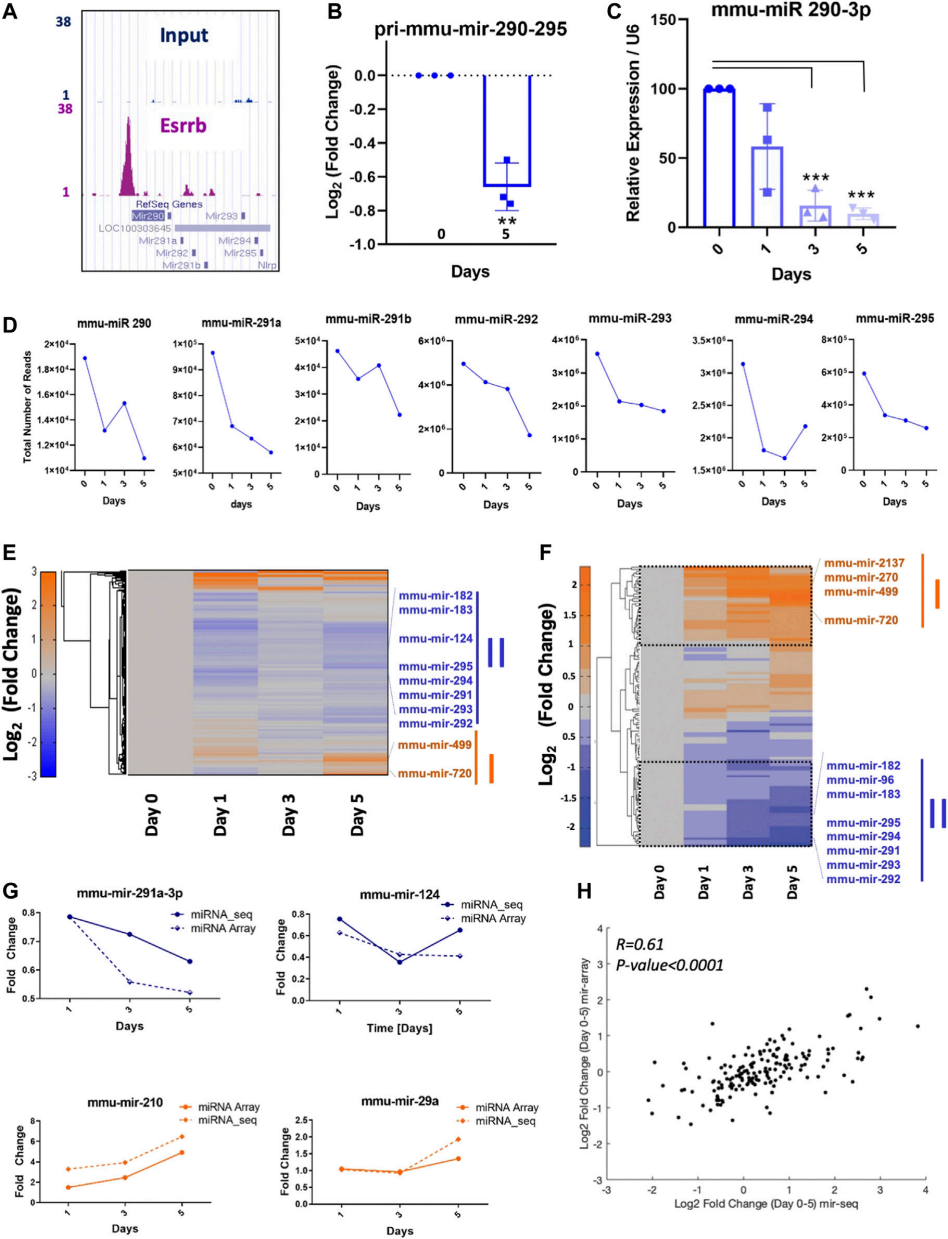
Direct regulation of miRNA expression by Esrrb **(A)** Genome Browser screenshot of the Esrrb binding site in the promoter region of the miR-290–295 cluster (http://genome.ucsc.edu ) (Kent et al., 2002). **(B)** Quantitative PCR measurements of changes in pri-mmu-mir-290–295 expression levels. Error bars indicate standard deviation derived from three independent time series. Significance was tested with a two-tailed Student´s *t*-test, with _**_ *p* < 0.001 **(C)** Relative expression of mature mmu-mir-290-3p over the time series. Error bars indicate standard deviation derived from three independent experiments (n = 3). Significance was tested comparing each day to day 0 using a two-tailed Student´s *t*-test with _***_ *p* < 0.0001) (D) Total number of reads for mmu-mir-290, mmu-mir-291a, mmu-mir-291b, mmu-mir-292, mmu-mir-293, mmu-mir-294, mmu-mir-295 at days d0, d1, d3 and d5. **(E)** Hierarchical clustering of the log_2_ (fold change) miRNA-Seq read counts after Esrrb knockdown. **(F)** Hierarchical clustering of the most significant miRNA level changes analysed by microarrays (Exiqon). **(G)** Similar expression profiles of miRNAs from the miRNA-Seq and the microarrary data. **(H)** Correlation plot between microRNAs obtained in microarrays and miRNA-Seq using the log2 of the fold change between day 0 and day 5. Correlation coefficient R = 0.61 with a *p*-Value *p* < 0.0001.

In mESC, the miR 290–295 cluster inhibits differentiation among other functions (Yuan et al., 2017). Analysis from our high-resolution Esrrb location analysis confirmed a clear peak within ≤5 Kb of the TSS on the miR 290–295 promoter (Figure 3A, Supplementary Table S2). Analysis of the primary miR-290–295 expression levels assessed by quantitative qPCR showed a significant downregulation of the primary transcript at day 5 (Figure 3B). In the same line, relative expression values of the mature mmu-miR-290-3p were significantly downregulated at day 3 and day 5 with respect to day 0 (Figure 3C). Effects on mature miR regulation were measured globally at day 0 in the presence of doxycycline and on days 1, 3 and 5 upon doxycycline removal using quantitative sequencing of short RNAs (18–30 nucleotides). Although, mature miRNAs might have long half-lives, a reduction in the mmu-mir-290, mmu-mir-291a, mmu-mir-291b, mmu-mir-292, mmu-mir-293, mmu-mir-294 and mmu-mir-295 was observed (Figure 3D and Supplementary Table S5). Clustering analysis of the miRNA-Seq data (Figure 3E) and the miRNA expression data analysed by the miRCURY LNA array platform (Figure 3F), showed comparable results for several miRNAs showing concordance in the downregulation or upregulation of many miRNAs (Figure 3G). This result was also confirmed by analyzing the correlation between the miRNAs obtained in the microarrays and the ones obtained in the miRNA-Seq analysis. Strong similarities were observed when the log_2_ fold change between day 0 and day 5 was compared (Figure 3H).

Esrrb is part a of mixed-type mRNA/microRNA feed-forward loop motif network that controls stem cell transition from pluripotency into early differentiation states.

**FIGURE 4:**
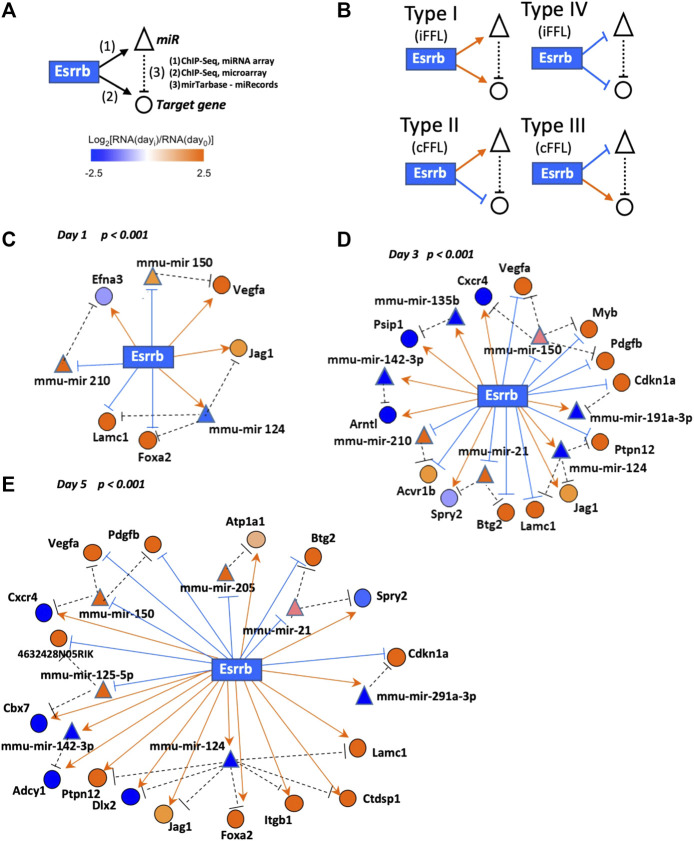
Esrrb regulated mRNA/miRNA feed-forward network motif. **(A)** Schematic view of the Esrrb regulated feed-forward network motif. Esrrb directly regulates expression of many target genes (1) as well as miRNAs (2), which in turn regulate expression levels of Esrrb protein-coding target genes (3). Interactions have been mapped according to experimentally validated post-translational regulations reported by either miRTarBase (last update January 2022) https://mirtarbase.cuhk.edu.cn/∼miRTarBase/miRTarBase_2022/php/index.php (Hsu et al., 2014) or miRecords (last update 03–09-2021) http://c1.accurascience.com/miRecords/(Xiao et al., 2009) databases. **(B)** Detected network motif types connecting Esrrb, miRNAs and their mRNA target genes. **(C–E)** Representation of the day 1, day 3 and day 5 networks linking together Esrrb, miRNA target genes and their experimentally validated mRNA targets. Esrrb is the central rectangle; circles and triangles designate protein-coding and miRNA-coding target genes, respectively. Edges are formatted either in bright orange arrows (activation) or blue hammerheads (repression). Nodes are coloured in orange or blue for both target genes and miRNAs, depending on their expression levels increase or decrease respectively, relative to day 0 levels. Only motifs that passed the Bonferroni corrected *p*-value (*p* < 0.001) during the Esrrb downregulation time course are depicted.

As many gene-products are regulated by miRNAs, the functions of potential regulatory network motifs containing both miRNAs and protein coding genes/gene products are gaining special interest (Zhang et al., 2015). For this reason, we focused our analysis on elucidating the presence of possible recurring Esrrb-controlled feed-forward loop network motifs (FFLs). These (FFL) motifs present three nodes: an upstream regulator X (Esrrb) that regulates both a downstream regulator Y (miR), and a downstream target Z (target-gene). An additional edge is directed from Y (miRNA) to Z (target-gene), thus closing the unidirectional “loop” (Figure 4A). FFLs can be divided into three types according to the master regulator: TF-FFL, miRNA-FFL and composite FFL in which TF and miRNA regulate each other (Shen-Orr et al., 2002; Zhang et al., 2013). Here we have considered the TF-FFLs where the TF (Esrrb) is the master regulator, which regulates its partner miRNA and their mutual target gene.

In our analysis, we found four types of feed-forward loop (FFL) motifs where Esrrb is the source transcriptional regulatory node by integrating, our previously published data of Esrrb genomic localization by Chip-Seq and, gene expression changes experimentally determined by the Affymetrix Gene 1.0 ST Array (Sevilla et al., 2021) with our miRNA expression changes experimentally analyzed in this study by miRCURY LNA™ miRNA Array followed by Esrrb downregulation (Supplementary Table S6). MiRNA/mRNA interacting pairs have been taken from the miRNA-target interactions experimentally validated databases miRTarBase https://mirtarbase.cuhk.edu.cn/∼miRTarBase/miRTarBase_2022/php/index.php (last update January 2022) (Hsu et al., 2014) or miRecords (last update 03–09-2021) http://c1.accurascience.com/miRecords/(Xiao et al., 2009) (Supplementary Table S9).

Based on the effects of TFs and miRNAs on their mutual target, FFLs can be classified in coherent and incoherent FFLs (Figure 4B). In incoherent FFLs, the expression of the target is controlled by two reverse paths, both miRNA and target gene interactions increase or decrease in expression together (Types I and IV), whereas in coherent FFLs, the regulatory paths have the same effects on the target (either activation or repression) and therefore, one interaction increase meanwhile the other decrease (Types II and III). In the case of coherent FFLs, the miRNA can help the transcriptional activation or repression of a target protein in the cell at a particular time acting as a post-transcriptional failsafe control whereas in the case of incoherent FFLs, the miRNA contributes more in fine-tuning the protein expression levels at the correct functional range (Osella et al., 2011; Zhang et al., 2013). Integration of the identified individual motifs revealed three networks corresponding to each day of the differentiation process with significant presence of specific Esrrb-regulated miRs (Figures 4C–E, Supplementary Table S9). As cells differentiate over time, more genes became differentially expressed and as a result, the subnetwork of FFL motifs grows. Although some of the miRNAs such as mmu-mir-150 and mmu-mir-124, are present in all the networks, the response is very dynamic as more targets are being affected by these miRNAs over time and because not all the targets are conserved over time. This effect is more evident between day 3 and day 5 were the number of targets increase and diversify. Only two miRNAs, mmu-miR-21 and mmu-miR-291-3p maintained the same targets from day 3 to day 5, whereas mmu-miR-135b appears only at day3.

Collectively, our data show that Esrrb controls FFLs involving both the protein coding target genes and miRNAs to guarantee the smooth transit from the pluripotency state into the early differentiation state in a precise controlled manner.

## Discussion

Gene expression regulation during the transition from pluripotency into the early stages of stem cell differentiation, is a complex process involving various regulatory biomolecules across different levels (MacNeil and Walhout, 2011). Transcription factors and miRNAs are the most common regulatory biomolecules that fine-tune gene expression by regulating at transcriptional and post-transcriptional level respectively (Martinez and Walhout, 2009). Although initially they were known to regulate gene expression independently, now increasing evidence shows that miRNAs and TFs also work synergistically in the form of complex networks to regulate the gene expression, which further modulates cellular and molecular processes (Qin et al., 2020; You et al., 2020; Bo et al., 2021). These complex regulatory interactions can be best viewed using TF-miRNA-Target Gene co-regulatory networks. These co-regulatory networks are responsible for the impressive degree of complexity in gene-regulation in higher eucaryotes (Cora et al., 2017).

Here we provide dynamic time series of miRNA significant changes after Esrrb downregulation in mouse ESCs (Figures 1D,E) and we connect this data with previously published high-resolution, genome-wide maps of Esrrb binding sites at promoter regions for most miRNA genes (Sevilla et al., 2021). From binding motif identification (Figure 2A), and changes in miRNA transcription, from the time series data generated in the present study (Figure 2D, Supplementary Tables S5, S6), we have identified the most significant Esrrb miRNAs target genes having a 57% of overlap with the previously identified Esrrb miRNA targets (Shao et al., 2015). Cross comparison of Esrrb miRNA targets with the other core transcription factors (Oct4, Sox2 and Nanog) miRNA targets, revealed twenty-nine miRNAs regulated uniquely by Esrrb. From them, 11 showed significant changes in their miRNA expression profiles upon Esrrb downregulation (Supplementary Table S6). Notably, those miRNA target genes that were also regulated by Esrrb as a transcription factor were target genes involved in signalling pathways related with pluripotency and negative regulation of apoptosis showing the complex regulatory mechanisms to maintain pluripotency (Figure 2I).

Among its direct targets, Esrrb regulates the miR 290–295 cluster (Figures 3A–D) as previously has been also proven to be regulated by other core transcription factors such as Oct4, Sox2, Nanog and Tcl3 (Marson et al., 2008). This cluster controls a wide variety of functions such as the regulation of expression of the core transcription factors, stem cell metabolism, cell proliferation and the cell cycle of ESCs whose phase distribution changes are critical for adequate stem cell differentiation (Yuan et al., 2017).

Additionally, special attention should be given to those miRNAs regulated by the core of transcription factors (OSNE) (Figure 2D). Among them we have the mmu-mir-182, which shows a clear upregulation by day 5 upon Esrrb downregulation in the absence of external signals. This upregulation is in line with recent studies where they show that the mmu-miR-182 and other differentially expressed miRNAs, act on neighborhoods of pluripotency genes to increase variation of target genes. Thus, through this mechanism, pluripotent stem cells could be driving cell diversification into new states without the need of external signals (Chakraborty et al., 2020).

Additionally, considering that Esrrb has a bimodal expression and that some of the differentially expressed miRNAs are regulated by Esrrb, the possibilities of cell diversification multiply exponentially since both mechanisms increase transcriptional variation (Kumar et al., 2014).

Our analyses of Esrrb-mediated miRNA gene regulation add a new element to the view of cell fate regulatory networks, a feed-forward control loop (FFL), consisted by Esrrb, direct miRNA targets and a protein coding gene transcriptionally regulated by Esrrb with further mRNA regulation by the miRNAs (Figures 4A,B). Our network studies show a novel approach to analyse miRNA expression dynamics during ESC differentiation, particularly at early stages when the cells transit from the pluripotency state into the differentiation fates.

Thoroughly observation of the connections between mRNA and miRNA-encoding genes, allowed the identification of frequently occurring feed-forward motifs composed of Esrrb as the central transcriptional regulatory node controlling the expression of mRNAs of certain genes as well as miRNAs that target these mRNAs (Figures 4C–E). Thus, from this data it appears that Esrrb and likely, other transcriptional regulators simultaneously control the expression of protein-coding genes as well as the miRNA-based machinery for post-transcriptional failsafe control or for fine-tuning protein levels. Through this mechanism cells presumably smoothly transit into early differentiation cell commitment.

ESCs exhibit a very unusual cell cycle structure, consisting mainly of an S phase and a short G1 phase, but lack of a G1/S checkpoint. In this study, downregulation of Esrrb elucidates a recurrent coherent motif type II (cFFL-type II) found at day 3 and day 5, formed by the mmu-mir-291a-3p and the target gene *Cdkn1a*. Previous studies lead by Blelloch and others have extensively characterize by luciferase reporter assays and qPCR the post-transcriptionally regulation of *Cdkn1a* by several members of the miR-290–295 cluster, in particular the mmu-mir-291a-3p, mmu-mir-291b-3p, mmu-mir-294 and mmu-mir-295 (Wang et al., 2008). Downregulation of mmu-mir-291a-3p abrogates the suppressing effect on *Cdkn1a,* an inhibitor of the cyclinE-Cdk2 complex. This mechanism slows down the cellular cell cycle for proper stem cell differentiation. Our data shows that, in this cell fate transition towards differentiation, the expression levels of this cell cycle inhibitor p21 (Cdkn1a) are post-transcriptionally regulated through an Esrrb-mmu-mir-291-3p-Cdkn1a coherent FFL type II motif acting as a failsafe control. Down-regulation of Esrrb reduces the expression of the mmu-mir-291a-3p which allows the expression of p21(Cdkn1a) (Supplementary Figure S3) (Wang et al., 2008).

In parallel, we have also observed several FFLs that could be involved in early stages of stem cell differentiation. One example is the FFL among Esrrb-mmu-mir-21 and Sprouty (Spry2), whose post-transcriptional regulation has been previously validated experimentally by luciferase reporter assays and western blot (Sayed et al., 2008; Mei et al., 2013). This Esrrb-mmu-mir-21-Spry2 FFL is present in our network as a coherent FFL type III (Figure 4 and Supplementary Figure S3). Previous studies have reported about this post transcriptional regulation of Spry2 by the mmu-mir-21 in cardiac and mesenchymal stem cell differentiation (Sayed et al., 2008; Mei et al., 2013).

Similarly, we have observed two other FFLs under the mmu-mir-124 regulating the expression of Lacm1 and the Notch ligand Jag1. A significant decrease in the luciferase activity for Jag1 has been observed in the presence of the mmu-mir-124 (Cheng et al., 2009) as well as a reduction of Lacm1 expression levels in the presence of mmu-mir-124 (Conaco et al., 2006). Both genes are known to regulate proliferation and self-renewal of neuronal stem cells (NSCs) (Stump et al., 2002; Cao et al., 2007). This finding supports previous reports where Esrrb depletion evidenced loss of pluripotency and certain cellular commitment towards neuroectoderm (Ivanova et al., 2006; Festuccia et al., 2018).

Finally, given that FFLs are generally not topologically isolated within the transcriptional regulatory network, they may be susceptible to; cross-talks among them, the transient dynamics of other regulatory modules, network motifs, or expressed proteins (Rowland et al., 2017). In this regard, our analysis considers the FFL networks at different days, as crucial events that control cell fate decisions, are likely to occur as regulatory networks process biological information in real time. Indeed, specific decisions may, in fact, be “emergent” properties of collective network dynamics. We hope that our novel systems biology approach and results will deeply influence future views and analyses of cell fate determination and more generally, the functions of biological regulatory networks.

## Conclusion

In summary, we have confirmed that TFs and miRNAs can jointly regulate target gene expression in the form of FFLs to transit from the pluripotency state into the early differentiation stages and that these TF-miRNA-Target Gene motifs are important genetic overrepresented motif patterns that occur more often than by chance in biological networks. Hence, FFLs in coregulatory networks are crucial in providing new insights into the logic and evolution of a new regulatory layer of the complex eukaryotic genome.

## Data Availability

The data presented in this study can be found in the online repository Gene Expression Omnibus (GEO) (Edgar et al., 2002; Barrett et al., 2011) under the accession numbers GSE57371, GSE189678 and the Esrrb ChiP-Seq data is under the accession numbers GSM785839 and GSM785840.
