# Supplementary figures and images for: Esrrb Regulates Specific Feed-Forward Loops to Transit From Pluripotency Into Early Stages of Differentiation
Source: Front Cell Dev Biol. 2022 May 16;10:820255. doi: 10.3389/fcell.2022.820255 (PMC9149258; doi:10.3389/fcell.2022.820255)

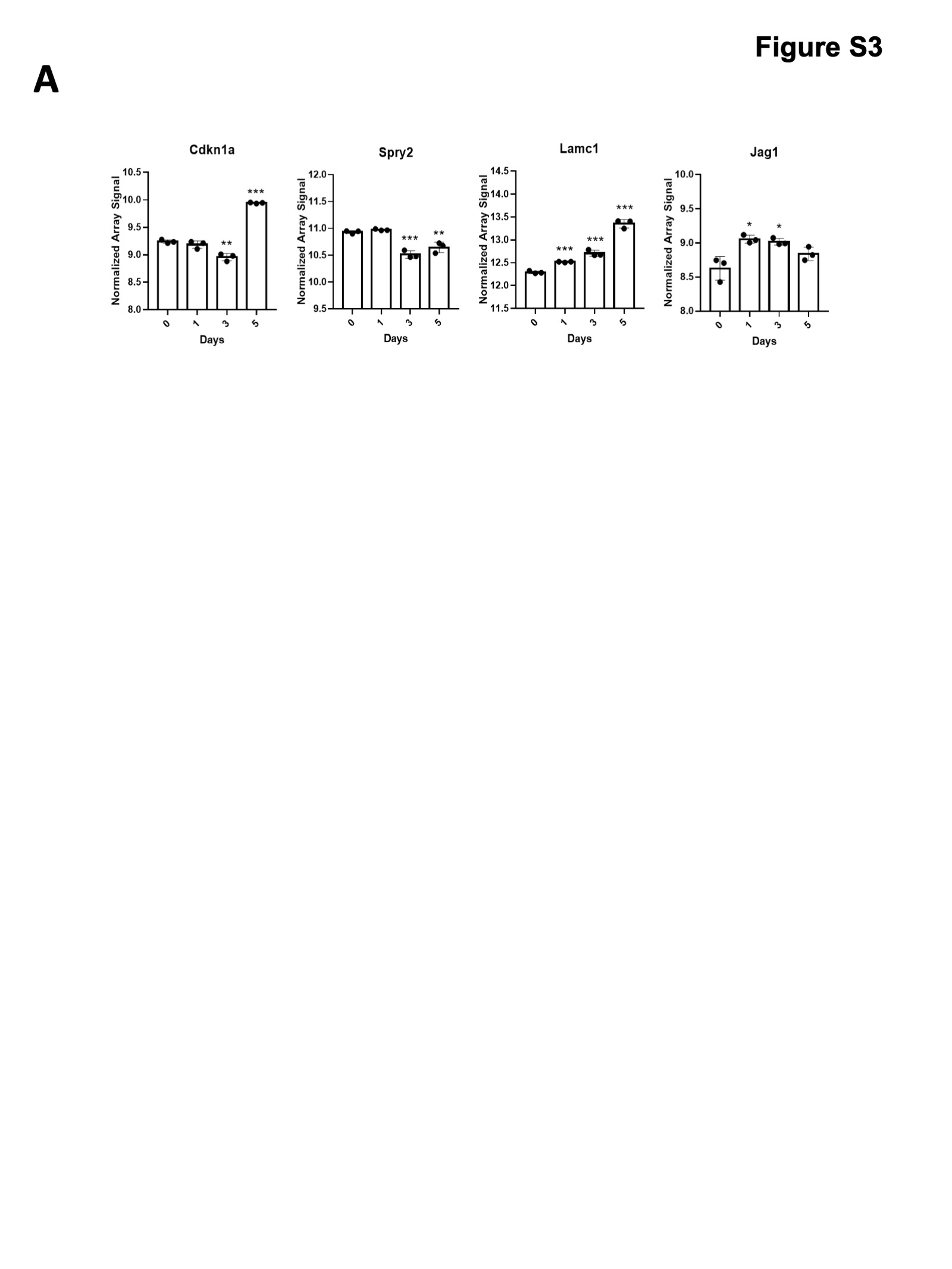

Supplement: Supplementary file 2 [file Image3.jpeg]

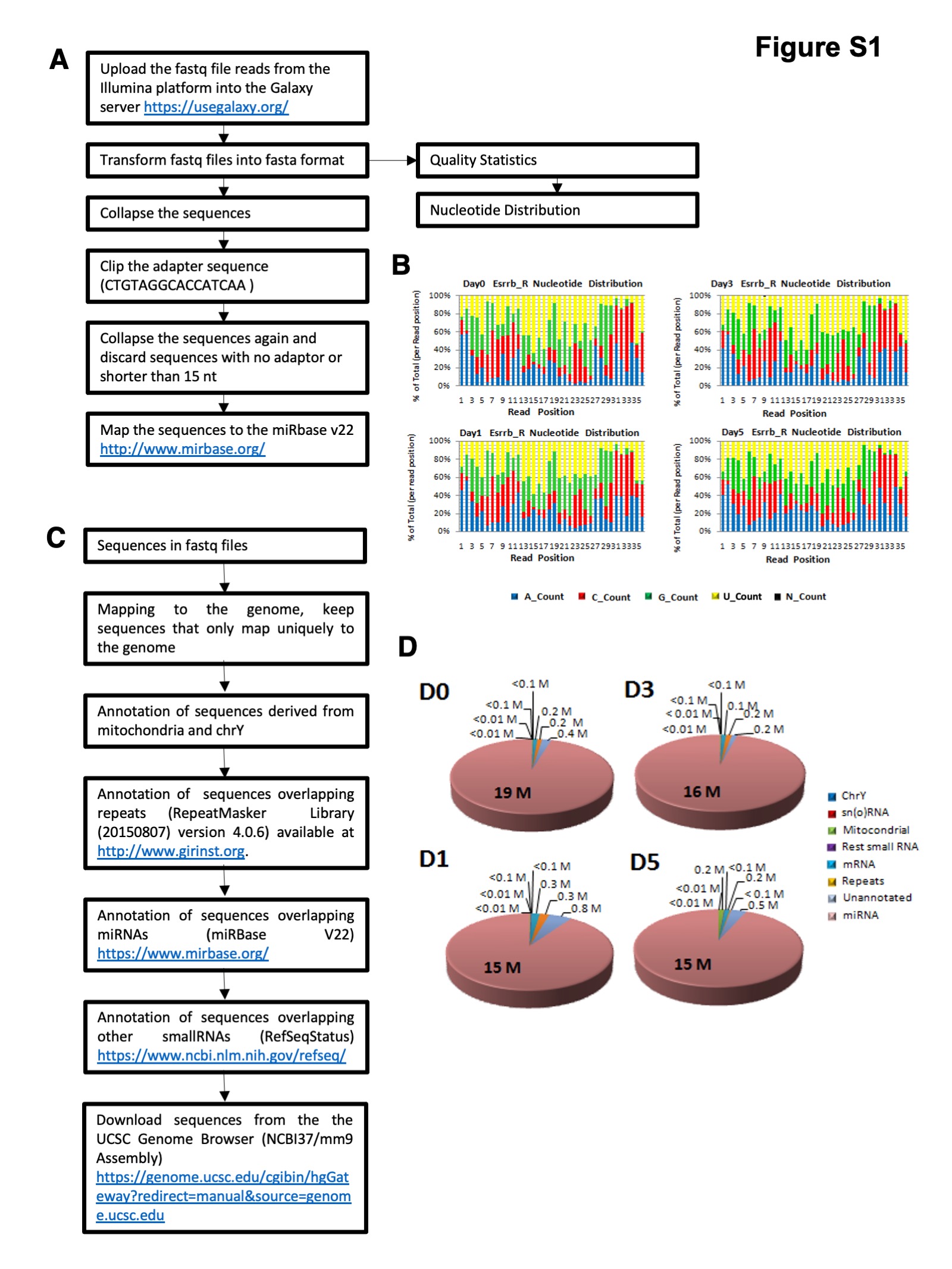

Supplement: Supplementary file 4 [file Image1.jpeg]

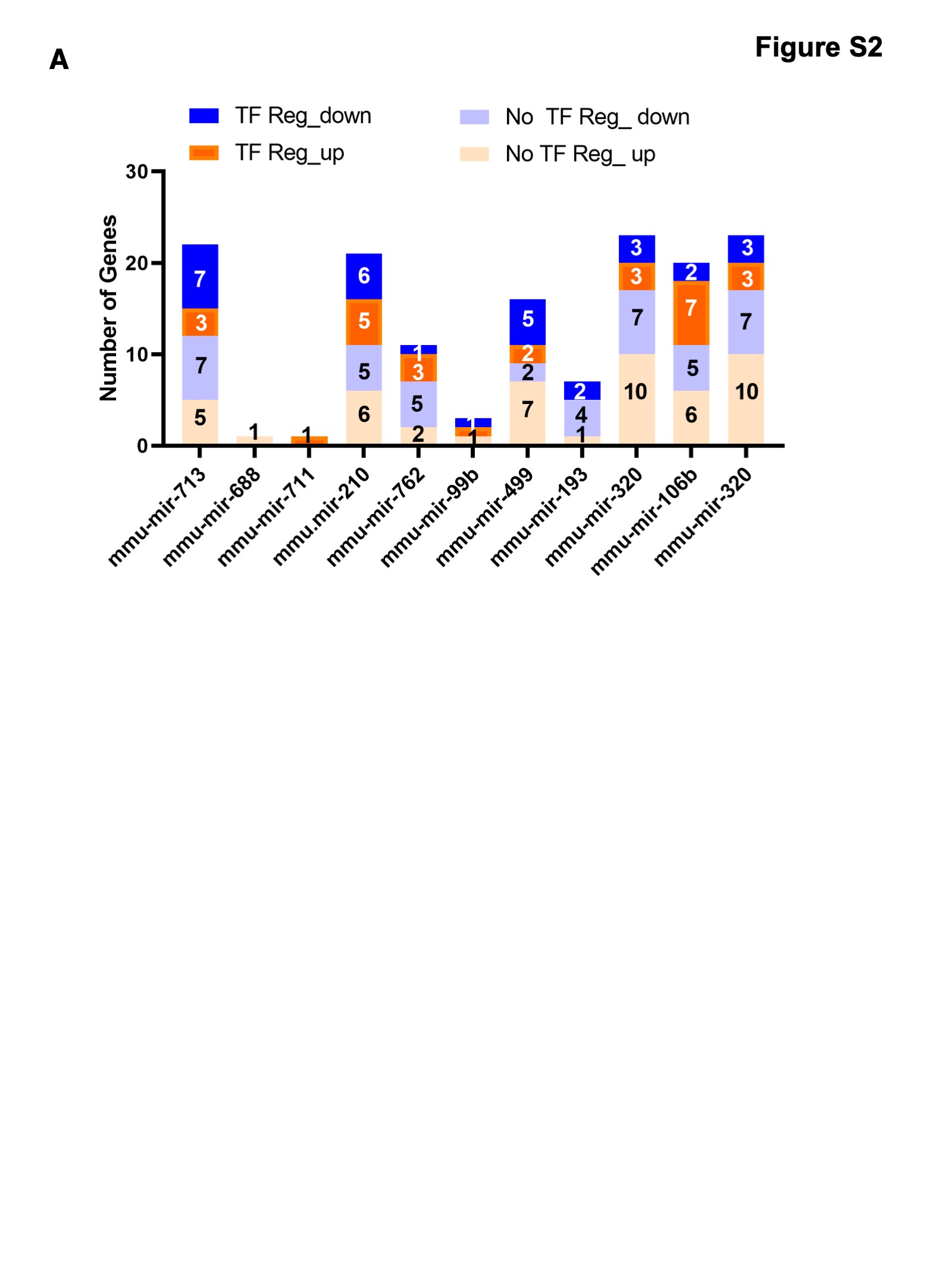

Supplement: Supplementary file 5 [file Image2.jpeg]
