# Supplementary material for: Esrrb Regulates Specific Feed-Forward Loops to Transit From Pluripotency Into Early Stages of Differentiation
Source: Front Cell Dev Biol. 2022 May 16;10:820255. doi: 10.3389/fcell.2022.820255 (PMC9149258; doi:10.3389/fcell.2022.820255)
Supplement: Supplementary file 8 [file DataSheet1.PDF]

Supplementary Material

Supplementary Material

Chromatin Immunoprecipitation

Data from the Esrrb ChIP was obtained from our previous work (Sevilla et al., 2021). Briefly, Esrrb chromatin immunoprecipitations (Esrrb-ChIP) was performed as described (Boyer et al., 2005). Images acquired from the Solexa sequencer were processed through the bundled Solexa image extraction pipeline and aligned to the Mouse July 2007 assembly (NCBI37/mm9) using ELAND software. Only uniquely mapped coordinates were used for further analyses. Uniquely aligned 36 bp sequences were extended to 250 bp in the 3' direction and allocated into 20 bp bins. We used a previously published algorithm (Zhang et al., 2008), Model-based Analysis of ChIP-Seq (MACS), for peak-finding. Binding regions were identified based on: (1) 250 bp bandwidth, (2) >10-fold and <30–fold change between total tag counts from each ChIP library vs. the negative/input ChIP library and (3) a p-value threshold of 10-5. Esrrb bound genomic DNA was enriched from whole cell lysates using anti-Esrrb antibody (R &D; PP-H6707-00, Clone H6707, Lot# A-1) and compared to the starting whole cell extracts. Complete sequence data is available at the NCBI GEO (Gene Expression Omnibus) database (Edgar et al., 2002; Barrett et al., 2011) under the accession designation numbers: GSM785839 and GSM785840.

MiRNA-Seq data analysis pipeline

All data analyses were performed on the NCBI37/mm9 assembly version of the mouse genome. Raw sequences files generated after image extraction and base-calling were pre-processed using the Galaxy software tools https://usegalaxy.org/ (Afgan et al., 2018). Sequences were imported in fastq files reads obtained from the Illumina platform after QC and pre-processing. Then fastq files were uploaded in the Galaxy platform https://usegalaxy.org/ to be transformed into fasta files (Figure S1A). Fasta files were tested for quality control (QC) statistics and nucleotide distribution visualization using FASTQC with default parameters (Figure S1B). Subsequently, sequences from the fasta files were collapse or grouped as unique after clipping the adaptor sequence CTGTAGGCACCATCAA. Sequences with no adaptor or shorter than 15 nucleotides were omitted from further analyses. A Perl program (Wall, L., Christiansen, T., & Orwant, 2000) was used to blat the sequences to the mirBase v22 http://www.mirbase.org/ (Kozomara and Griffiths-Jones, 2014; Kozomara et al., 2019) using the command line: blat -tileSize=6 -stepSize=5 -minScore=16 -minIdentity=60 hairpin.fa. The relative frequency of miRNAs was determined by normalizing miRNA reads against the total count of 18–22 nucleotide reads (Linsen et al., 2009; Git et al., 2010). Normalized data have been reported as reads (or transcripts) per million for each respective library (Table S**5**). In summary, sequencing depth of the raw sequences for day 0 was 29,388 millions of reads, and 26,431 millions of sequences were mapped

to the miRbase v22 (www.mirbase.org), for day1 we obtained 26,171 millions of raw sequences and 23,647 millions were mapped, for day 3 we obtained 27,912 millions of raw sequences and 24,505 millions were mapped and for day 5 we obtained 28,532 millions of raw sequences and 26,146 millions were mapped (Table S5). Figure S1A-C depicts the steps followed for the miRNA sequencing data analysis. All files obtained from the analysis using the Galaxy software, are at this github repository https://github.com/angelcroman/esrrb_mirna. From the Fastq files, we also applied the Bowtie program to map sequences to the mouse genome (Langmead et al., 2009). Sequences that were perfectly and uniquely mapped on the genome were kept for further annotations. Some of the annotations used for filtrating small RNAs (sRNAs) such as repeat regions, annotated sno- and miRNAs and predicted RNA genes were obtained from the following UCSC browser tracks: Repeat Masker, sno/miRs, RNA genes, miRNA, RefGene, RefSeq Status (O'Leary et al., 2016) and were downloaded from the UCSC Genome Browser (http://genome.ucsc.edu) (NCBI37/mm9 Assembly) (Kent et al., 2002), Figure S1D (see file Esrrb_PD_6_miRNAs_Seq_Summary_of_mapping_and_annotation at https://github.com/angelcroman/esrrb_mirna).

miRNA Microarray expression data analysis

Median signal intensities for all microarray probes were background subtracted and tabulated. The data were then quantile normalized by assigning each probe the average signal intensity for all probes of the same intensity rank across the 12 experiments. Signal intensities were then floored at one unit and log transformed. Control probes were removed from further analysis. We next identified miRNA probes that were differentially enriched in day 0 and the other time points. Statistically significant differential expression for the different samples was calculated using one-way ANOVA (Ayroles and Gibson, 2006) and Bonferroni correct p-value of 0.05. Out of 332 probes, 125 were determined to be differentially expressed at least at one time point (Table S6).

Establishing a genome –wide TF-miRNA-mRNA FFL network

Regulatory Network Motif

Three types of regulatory associations were used to construct a comprehensive TF-miRNA-mRNA-FFL network. First, the TF-mRNA associations were obtained from our Esrrb Chip-Seq data analyses considering those gene targets in the vicinity (≤5Kb) of the promoter region or within a distal functional binding site region of a target gene. Target gene expression profiles were taken from the Esrrb downregulation time series microarray data previously published (Sevilla et al., 2021). The TF-miRNA associations were obtained from our Esrrb Chip-Seq data analysis considering those targets in the promoter or the distal functional region of a microRNA gene. MiRNA expression profiles were taken from the microarray data generated in this study after Esrrb downregulation (Table S6).

Finally, experiments supporting the miRNA-gene regulatory association were obtained from post-transcriptionally regulations experimentally validated collected in the miRTarBase (last update from Jan 2022) https://mirtarbase.cuhk.edu.cn/~miRTarBase/miRTarBase_2022/php/index.php (Huang et al., 2020) or miRrecords (last update 03-09-2021) http://c1.accurascience.com/miRecords/ (Xiao et al., 2009). Records of the experimental miRNA gene target interactions by either Luciferase reporter assay, western-blot or qPCR, are depicted in Table S9 with the corresponding PubMed accession numbers.

In summary, each motif is composed of exactly three nodes: the transcription factor (Esrrb, which is common to all motifs), a microRNA that is regulated by Esrrb, and a target gene that is reported to be regulated by both, the TF (Esrrb) and the microRNA regulated by Esrrb. And each module has three edges, where each of them is characterized by the regulatory effect of the source of the target. Therefore, two edges that connect the TF node to the target miRNA and target gene that can be an arrow or a spate edge depending on the type of regulation, and a third edge that is always an spate edge as it connects the miRNA to the target gene (Fig 4A).

Esrrb Gene Targets

Esrrb gene targets were selected from our Esrrb Chip-Seq data (Sevilla et al., 2021).

Esrrb miRNA Targets

miRNA targets for *Esrrb* were selected based on enrichment peaks from our Esrrb Chip-Seq experiment, containing both gene-distal loci peaks and promoter peaks at miRNA genomic sequences (Sevilla et al., 2021). Among the Chip-Seq peaks that were significantly higher than the background (Table S2), there were 238 miRNA genomic sequences that existed in the vicinity of promoter region or distal regions where the significant peaks were found, and those microRNA were adopted as Esrrb targets.

miRNA Gene Targets

MiR/mRNA interacting pairs have been taken from the miR-target interactions experimentally validated databases miRTarBase (last update from Jan 2022) https://mirtarbase.cuhk.edu.cn/~miRTarBase/miRTarBase_2022/php/index.php (Hsu et al., 2014) or miRecords (last update 03-09-2021) http://c1.accurascience.com/miRecords/ (Xiao et al., 2009) (Table S9).

Statistical Tests

In order to identify genes and miRNAs that were significantly changed on each day with respect to day 0, two tail t-test was applied on each data sets and significant levels were corrected with a Bonferroni factor, accordingly. For determining the genes and miRNA that experience at least a change across three time points. Initial ANOVA test was applied on each dataset, an on each case results were subjected Bonferroni correction.

Δ Normalizing Log Mean Expression Level (ΔNormalized LME)

In order to calculate the difference in expression levels of a motif node (i.e. TF, miRNA, or Gene) between day *i* and day 0, that were identified to be statistically different, we calculated a scaled vector of the Log_2 Expression levels of a component centered around 0 and subtract the day 0 component from day *i*.

Partial Correlation

Partial correction between MicroRNA and gene controlling for *Esrrb* was calculated using Eq. (1)

$$\rho_{(xy \cdot z)} = \frac{\rho_{xy} - \rho_{xz} \cdot \rho_{yz}}{\sqrt{(1-\rho_{xz}^2)(1-\rho_{yz}^2)}} \quad (1)$$

In the above equation ρ_{xy} ,ρ_{xz} , and ρ_{yz} are the Pearson correlation Coefficients between expression levels: a miRNA and gene, miRNA and Esrrb, and gene and miRNA across all four time points (days 0,1,3, and 5), respectively. The interpretation of the partial correction is based on discussion in (Cramer, 2003).

Bioinformatic analyses to generate the networks shown in (Figure 4 C-E) were done using the most recent version of the R software version 4.1.2 (R Core Team, 2021) through Rstudio 1.4.1103 (R Studio Team, 2020). The scripts in R were used to properly compute and identify the relationship of Esrrb with each gene and miRNA, by finding out whether the knock-down of Esrrb implies a significant change in the expression of those genes or miRs. Regulatory networks of TF-miRNA–mRNA interactions were constructed and visualized using in Cytoscape version 3.8.2 (http://www.cytoscape.org/ (accessed on 20 January 2022)(Gustavsen et al., 2019) where we were able to visualize the dynamics of the different types of motifs establishing a gene color gradient according to their previously computed gene expression profiles.

Supplemental Figure Legends

Figure S1. Pipeline for miRNA sequencing data analysis. Short RNA annotation processing pipeline. (A) Simple representation of the steps applied to short RNA sequences is shown using https://usegalaxy.org/ (Afgan et al., 2018). (B) Stacked-histogram graph showing the nucleotide sequence distribution in the Solexa library. Adenine counts represented in blue, cytosine counts in red, guanine counts in green, and uracil counts in yellow. Non identified nucleotides are represented in black (C) Pipeline applied to sRNA sequence annotation. (D) Genomic distribution of sRNAs at different days from Fastq files. Pie charts represent the distribution of the eight different classes of sRNAs measured. 'Rest small RNA' represents UCSC database annotated sRNAs filtered from mitochondria, the Y-chromosome, repeats, mRNAs, miRs, snRNAs and snoRNAs. 'Unannotated'

represents sRNAs that have no information in the UCSC database. Total number of reads were mapped to the mouse reference genome (July 2007, NCBI37/mm9).

Figure S2. MiRNA targets regulated by unique significant Esrrb regulated miRNAs. Dark orange and dark blue represent the set of gene targets upregulated or downregulated which are both directly regulated by Esrrb as transcription factor (≤ 5Kb from TSS) and regulated by Esrrb through the regulation of miRNAs. Light orange and light blue represent the set of gene targets upregulated or downregulated which are only regulated by Esrrb through the modulation of miRNAs.

Figure S3. Expression levels of Cdkn1a, Spry2, Lamc1 and Jag1. Affymetrix Gene Chip Mouse Gene 1.0 microarrays were probed with RNA from samples harvested at day 0, 1, 3 and 5. Shown are the normalized array signals for Cdkn1a, an inhibitor of the transition from G1 to S induced by the cyclin E–Cdk2 complex, Spry2, Lamc1and Jag1 Results are shown as means ± s.d.; (n =3). Significance was tested comparing each day to day 0 using a two-tailed Student´s *t*-test with *** *P* <0.0001, ** *P* <0.001 and * *P* <0.01.

REFERENCES

Afgan, E., Baker, D., Batut, B., Van Den Beek, M., Bouvier, D., Ech, M., et al. (2018). The Galaxy platform for accessible, reproducible and collaborative biomedical analyses: 2018 update. *Nucleic Acids Res.* 46, W537–W544. doi:10.1093/NAR/GKY379.

Ayroles, J. F., and Gibson, G. (2006). [11] Analysis of Variance of Microarray Data. *Methods Enzymol.* 411, 214–233. doi:10.1016/S0076-6879(06)11011-3.

Barrett, T., Troup, D. B., Wilhite, S. E., Ledoux, P., Evangelista, C., Kim, I. F., et al. (2011). NCBI GEO: Archive for functional genomics data sets-10 years on. *Nucleic Acids Res.* 39. doi:10.1093/nar/gkq1184.

Boyer, L. A., Tong, I. L., Cole, M. F., Johnstone, S. E., Levine, S. S., Zucker, J. P., et al. (2005). Core transcriptional regulatory circuitry in human embryonic stem cells. *Cell* 122, 947–956. doi:10.1016/j.cell.2005.08.020.

Cramer, D. (2003). A cautionary tale of two statistics: partial correlation and standardized partial regression. *J. Psychol.* 137, 507–511. doi:10.1080/00223980309600632.

Edgar, R., Domrachev, M., and Lash, A. E. (2002). Gene Expression Omnibus: NCBI gene expression and hybridization array data repository. *Nucleic Acids Res.* 30, 207–210. doi:10.1093/nar/30.1.207.

Friedman, R. C., Farh, K. K. H., Burge, C. B., and Bartel, D. P. (2009). Most mammalian mRNAs are conserved targets of microRNAs. *Genome Res.* 19, 92–105. doi:10.1101/GR.082701.108.

Git, A., Dvinge, H., Salmon-Divon, M., Osborne, M., Kutter, C., Hadfield, J., et al. (2010). Systematic comparison of microarray profiling, real-time PCR, and next-generation sequencing technologies for measuring differential microRNA expression. *RNA* 16, 991–1006. doi:10.1261/rna.1947110.

Gustavsen, J. A., Pai, S., Isserlin, R., Demchak, B., and Pico, A. R. (2019). RCy3: Network biology using Cytoscape from within R. *F1000Research* 8, 1774. doi:10.12688/F1000RESEARCH.20887.2.

Huang, H. Y., Lin, Y. C. D., Li, J., Huang, K. Y., Shrestha, S., Hong, H. C., et al. (2020). miRTarBase 2020: updates to the experimentally validated microRNA-target interaction database. *Nucleic Acids Res.* 48, D148–D154. doi:10.1093/NAR/GKZ896.

Kent, W. J., Sugnet, C. W., Furey, T. S., Roskin, K. M., Pringle, T. H., Zahler, A. M., et al. (2002). The Human Genome Browser at UCSC. *Genome Res.* 12, 996–1006. doi:10.1101/GR.229102.

Kozomara, A., Birgaoanu, M., and Griffiths-Jones, S. (2019). miRBase: from microRNA sequences to function. *Nucleic Acids Res.* 47, D155–D162. doi:10.1093/NAR/GKY1141.

Kozomara, A., and Griffiths-Jones, S. (2014). miRBase: annotating high confidence microRNAs using deep sequencing data. *Nucleic Acids Res.* 42. doi:10.1093/NAR/GKT1181.

Langmead, B., Trapnell, C., Pop, M., and Salzberg, S. L. (2009). Ultrafast and memory-efficient alignment of short DNA sequences to the human genome. *Genome Biol.* 10. doi:10.1186/gb-2009-10-3-r25.

Linsen, S. E. V., de Wit, E., Janssens, G., Heater, S., Chapman, L., Parkin, R. K., et al. (2009). Limitations and possibilities of small RNA digital gene expression profiling. *Nat. Methods* 6, 474–476. doi:10.1038/nmeth0709-474.

O'Leary, N. A., Wright, M. W., Brister, J. R., Ciufo, S., Haddad, D., McVeigh, R., et al. (2016). Reference sequence (RefSeq) database at NCBI: current status, taxonomic expansion, and functional annotation. *Nucleic Acids Res.* 44, D733–D745. doi:10.1093/NAR/GKV1189.

R Core Team (2021). R: A language and environment for statistical computing. R Foundation for Statistical Computing. *Vienna. Austria*. Available at: https://www.r-project.org/.

R Studio Team (2020). RStudio: Integrated Development for R. RStudio, PBC,. *Boston, MA*. Available at: http://www.rstudio.com/.

Sevilla, A., Papatsenko, D., Mazloom, A. R., Xu, H., Vasileva, A., Unwin, R. D., et al. (2021). An Esrrb and Nanog Cell Fate Regulatory Module Controlled by Feed Forward Loop Interactions. *Front. Cell Dev. Biol.* 9, 630067–630067. doi:10.3389/fcell.2021.630067.

Wall, L., Christiansen, T., & Orwant, J. (2000). Programming perl.

Xiao, F., Zuo, Z., Cai, G., Kang, S., Gao, X., and Li, T. (2009). miRecords: An integrated resource for microRNA-target interactions. *Nucleic Acids Res.* 37. doi:10.1093/nar/gkn851.

Zhang, X., Zhang, J., Wang, T., Esteban, M. A., and Pei, D. (2008). Esrrb activates Oct4 transcription and sustains self-renewal and pluripotency in embryonic stem cells. *J. Biol. Chem.* 283, 35825–35833. doi:10.1074/jbc.M803481200.
